# Pharmacological characterization of the antidiabetic drug metformin in atherosclerosis inhibition: A comprehensive insight

**DOI:** 10.1002/iid3.1346

**Published:** 2024-08-02

**Authors:** Areej Turkistani, Haydar M. Al‐Kuraishy, Ali I. Al‐Gareeb, Athanasios Alexiou, Marios Papadakis, Mostafa M. Bahaa, Salah Al‐Windy, Gaber El‐Saber Batiha

**Affiliations:** ^1^ Department of Pharmacology and Toxicology, College of Medicine Taif University Taif Saudi Arabia; ^2^ Department of Clinical Pharmacology and Medicine, College of Medicine Mustansiriyah University Baghdad Iraq; ^3^ Department of Clinical Pharmacology and Medicine Jabir ibn Hayyan Medical University Kufa Iraq; ^4^ Department of Science and Engineering Novel Global Community Educational Foundation Hebersham New South Wales Australia; ^5^ AFNP Med Wien Austria; ^6^ Department of Research & Development Funogen Athens Greece; ^7^ University Centre for Research & Development Chandigarh University Punjab India; ^8^ Department of Surgery II, University Hospital Witten‐Herdecke University of Witten‐Herdecke Wuppertal Germany; ^9^ Pharmacy Practice Department, Faculty of Pharmacy Horus University New Damietta Egypt; ^10^ Department of Biology, College of Science Baghdad University Baghdad Iraq; ^11^ Department of Pharmacology and Therapeutics, Faculty of Veterinary Medicine Damanhour University Damanhour Egypt

**Keywords:** atherosclerosis, Kruppel like factor 2, metformin, nuclear erythroid related factor 2

## Abstract

**Background:**

Atherosclerosis (AS) is a progressive disease that interferes with blood flow, leading to cardiovascular complications such as hypertension, ischemic heart disease, ischemic stroke, and vascular ischemia. The progression of AS is correlated with inflammation, oxidative stress, and endothelial dysfunction. Various signaling pathways, like nuclear erythroid‐related factor 2 (Nrf2) and Kruppel‐like factor 2 (KLF2), are involved in the pathogenesis of AS. Nrf2 and KLF2 have anti‐inflammatory and antioxidant properties. Thus, activation of these pathways may reduce the development of AS. Metformin, an insulin‐sensitizing drug used in the management of type 2 diabetes mellitus (T2DM), increases the expression of Nrf2 and KLF2. AS is a common long‐term macrovascular complication of T2DM. Thus, metformin, through its pleiotropic anti‐inflammatory effect, may attenuate the development and progression of AS.

**Aims:**

Therefore, this review aims to investigate the possible role of metformin in AS concerning its effect on Nrf2 and KLF2 and inhibition of reactive oxygen species (ROS) formation. In addition to its antidiabetic effect, metformin can reduce cardiovascular morbidities and mortalities compared to other antidiabetic agents, even with similar blood glucose control by the Nrf2/KLF2 pathway activation.

**Conclusion:**

In conclusion, metformin is an effective therapeutic strategy against the development and progression of AS, mainly through activation of the KLF2/Nrf2 axis.

## 1 INTRODUCTION

Atherosclerosis (AS) is a condition characterized by fatty deposition in the inner wall, focal thickening of the arterial wall with formation of atherosclerotic plaques.^1^ It is a progressive disease that interferes with blood flow resulting in stroke and ischemic heart disease.^2^, ^3^ The femoral, vertebral, basilar, cerebral, and coronary arteries are the major areas affected by AS. The main reasons for death from AS include peripheral vascular disease, stroke, and myocardial infarction.^4^ Surprisingly, the AS process may begin in childhood but only become clinically apparent in middle life and later on.^5^, ^6^, ^7^

Cardiovascular complications are mostly caused by atherosclerotic plaque rupture and related thromboembolic diseases.^8^ Inflammation, oxidative stress, endothelial dysfunction, apoptosis, vascular proliferation, matrix degeneration, and neovascularization are the fundamental underlying pathological conditions related to the progression of AS.^9^, ^10^ Dyslipidemia, particularly hypercholesterolemia is thought to be the primary inducer of AS because it increases endothelial permeability, which makes it easier for lipid particles to enter and deposit in the vascular endothelium.^11^ It is believed that lipid particles, particularly LDL, in the subendothelial space serve as chemoattractants for monocytes, which then develop into foamy macrophages.^12^ Additionally, oxidized LDL in the subendothelial space causes macrophage scavenger receptor expression and further intracellular cholesterol buildup.^12^

These pathological alterations encourage plaque development, vascular lumen narrowing, and AS development. A higher infiltration of T cells into atherosclerotic plaques increases their susceptibility to rupture and thrombosis.^13^ Atherosclerotic plaques are vulnerable to erosion, rupture, and calcification with the development of nodules.^14^, ^15^ The onset of premature AS is thought to be strongly predicted by high LDL, TG, and low HDL. High HDL levels are thought to be protective against the onset and worsening of AS. Additionally, hypertriglyceridemia is regarded as a separate risk factor for AS development. Likewise, AS pathophysiology is connected to abnormalities of the lipoproteins. Elevated lipoprotein A is associated with the development of AS.^16^, ^17^ Notably, macrophages are the most important immune cells involved in the progression of AS and atherosclerotic complications, including erosion and rupture. Normally, immune cells, mainly macrophages, consume oxidized LDL (ox‐LDL) and produce reactive oxygen species (ROS). In turn, excessive production of ROS promotes the development of oxidative stress and the progression of plaque instability. As a result, ox‐LDL accelerates macrophage oxidative stress processes as oxidative stress progresses.^18^, ^19^

Oxidative stress and inflammation enhance the progression of AS in a vicious cycle. As well, inflammation induces the development and progression of oxidative stress and vice versa.^19^ Oxidative stress promotes the expression of inflammatory signaling pathways and produces proinflammatory cytokines, and chemokines, which in turn enhance ROS generation.^19^ NADPH‐oxidase is a widely expressed enzyme, mainly in vascular smooth muscle, involved in ROS generation. Higher expression of NADPH‐oxidase is increased by the aging process, leading to endothelial dysfunction, vascular inflammation, and mitochondrial and cellular‐induced oxidative stress.^20^

**Figure 1:**
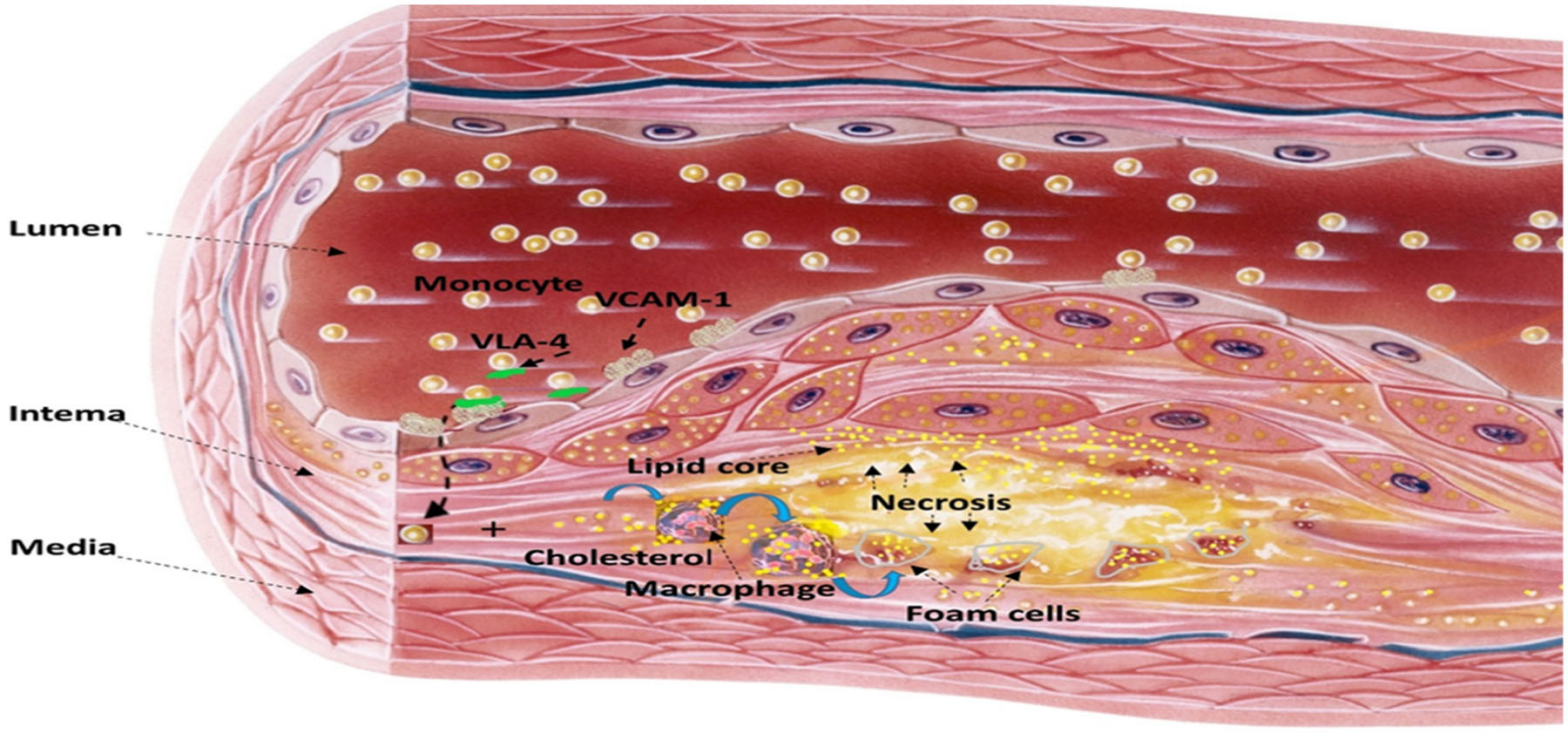
Pathophysiology of atherosclerosis: Monocyte via very late antigen 4 (VLA4) binds vascular cell adhesion molecule 1 (VCAM‐1) and enter the vascular lumen, then converted to macrophages which uptake cholesterol and converted to foam cells that undergo necrosis and deliver lipid into the lipid core with formation of atherosclerotic plaque.

Ox‐LDL has been demonstrated to stimulate monocyte infiltration and smooth muscle cell migration. Through the stimulation of endothelial cell apoptosis, plaque erosion, production of tissue factors, and disruption of the endogenous anticoagulant pathway, it contributes to atherothrombosis. In a physiologically healthy state, HDL reduces the generation and effects of ox‐LDL.^21^, ^22^ Furthermore, oxidized HDL (ox‐HDL) loses its vasculoprotective effect and is thought to be proinflammatory and proatherogenic, thereby increasing the risk of AS progression. Notably, ox‐HDL promotes the progression of atherosclerotic plaque erosion and rupture. Therefore, ox‐HDL is regarded as a potential risk factor for AS and atherothrombosis.^23^ These findings demonstrated that the pathogenesis of AS is a multifactorial process involving dyslipidemia, related inflammatory diseases, and oxidative stress (Figure 1).

Different signaling pathways, including nuclear erythroid‐related factor 2 (Nrf2) and Kruppel‐like factor 2 (KLF2), are intricate in the pathogenesis of AS.^24^ Nrf2 is a transcription factor that mediates various biological functions, including oxidative stress and drug metabolism.^24^ Nrf2 in response to oxidative stress increases the expression of glutathione–S‐transferase (GST) and other antioxidant enzymes.^25^ As well, KLF2 is a mechano‐sensitive transcription factor involved in the regulation of endothelial functions. In addition, KLF2 has anti‐inflammatory and antioxidant effects, preventing the progression and development of endothelial dysfunction. Therefore, activation of these pathways by specific activators may reduce the progression and development of AS. Notably, metformin, an insulin‐sensitizing drug used as first‐line treatment for type 2 diabetes mellitus (T2DM), improves Nrf2 and KLF2 expression.^26^

It has been reported that dysregulation of insulin signaling and the development of insulin resistance (IR) is implicated in the pathogenesis of AS.^27^ An important distinction to draw is the role of IR versus hyperglycemia in the development of AS. Although both likely have a synergistic atherogenic effect in the setting of T2DM, IR has been shown to have a strong link to cardiovascular disease, even in the absence of hyperglycemia.^27^ IR promotes a proinflammatory state and dyslipidemia in addition to perturbed insulin signaling on important intimal cells (endothelial, vascular smooth muscle cells, and macrophages) resulting with advanced plaque progression in the setting of hyperinsulinemia.^28^ In addition, insulin has several pleiotropic effects, such as anti‐inflammatory, antithrombotic, and antioxidant properties. Insulin, per se, exerts an inhibitory effect on the activation of oxidative stress and seems able to counteract the pro‐oxidant effects of ambient hyperglycemia and glycaemic variability. However, insulin actions remain a subject of debate with respect to the risk of adverse cardiovascular disease events, which can increase in individuals exposed to high insulin doses.^29^ Therefore, modulation of insulin signaling by insulin sensitizing agents could be effective in the management of AS. Notably, metformin, an insulin‐sensitizing drug used as first‐line treatment for T2DM, improves Nrf2 and KLF2 expression.^30^ Therefore, this review aims to discuss the potential role of metformin in AS concerning its effect on Nrf2 and KLF2 signaling pathways.

## 2 METFORMIN CHEMISTRY, PHARMACOLOGY, AND PHARMACODYNAMICS

**Figure 2:**
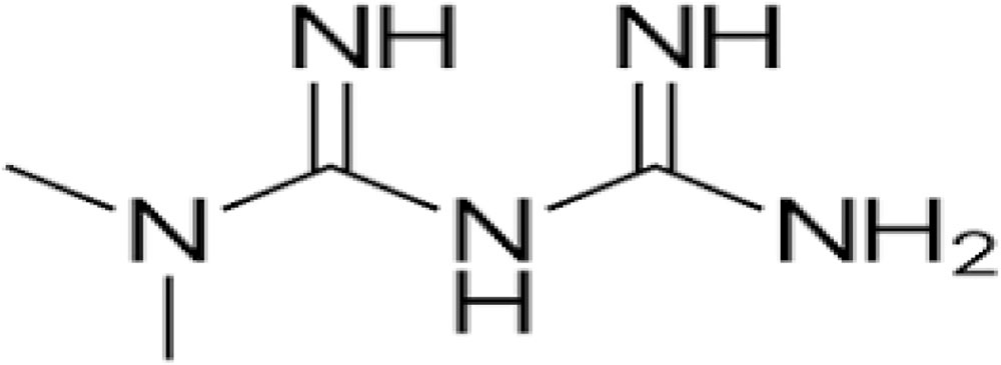
Chemical structure of metformin.

Metformin belongs to the biguanide group and has the ability to reduce peripheral IR. Metformin is 3‐(diaminomethylidene)‐1,1‐dimethylguanidine^31^ (Figure 2).

Metformin is an orally active drug that is absorbed from the small intestine by the plasma membrane monoamine transporter, which is highly expressed in the enterocytes. Organic cation transporter 2 (OCT2), which is expressed on the brush border of enterocytes, is involved in the uptake of metformin.^32^ However, the roles of OCT1 and OCT3 were not identified. OCT1 has the largest influence in the liver's ability to absorb metformin, with OCT3 playing a minor contribution. Metformin absorption by renal epithelial cells is dependent on the protein OCT2. Metformin excretion by the kidney occurs through multidrug and toxin extrusion 1 (MATE1). Metformin is not metabolized and is excreted unchanged by the kidney. Metformin has a half‐life of 5 h, is widely distributed and has a plasma steady‐state concentration ranging from 54 to 4133.^33^

**Figure 3:**
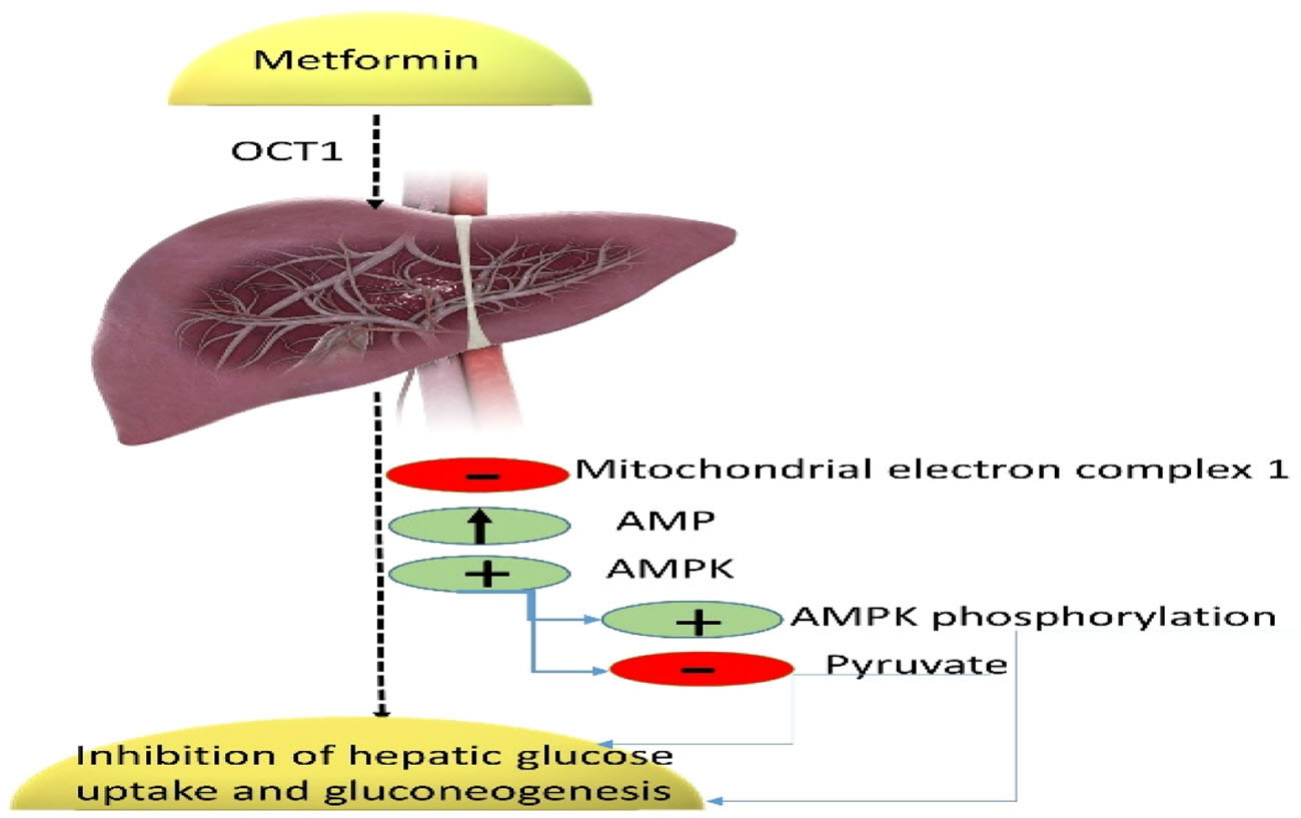
Pharmacodynamics of metformin: Metformin uptake by the liver is through organic cation transporter 1 (OCT1), which inhibits mitochondrial electron Complex 1 and promotes activation of adenosine monophosphate protein kinase (AMPK), leading to inhibition of hepatic glucose uptake and gluconeogenesis.

Metformin has a unique pharmacodynamic effect, and after uptake by OCT2 in the small intestine, it is highly absorbed. Metformin is a positive charge molecule; it highly accumulates in the cells, mainly in the mitochondria, because of the negative charge of the mitochondrial membrane. It is extensively taken up by OCT1 in hepatocytes, where it has the greatest pharmacological effect.^34^, ^35^ Metformin inhibits ATP production through inhibition of mitochondrial Complex I, leading to an increase in AMP‐ATP with an increasing level of adenosine monophosphate protein kinase (AMPK). AMPK inhibits gluconeogenesis, fat synthesis, and hepatic fat storage while improving insulin sensitivity and anaerobic glucose metabolism in the enterocytes. As well, metformin reduces hepatic lactate uptake while increasing lactate delivery into circulation.^36^, ^37^ Metformin promotes glucose utilization by the gut microbiota by activating the release of glucagon‐like peptide‐1 (GLP‐1) from L cells in the intestine, and it improves peripheral glucose utilization by increasing the expression of the glucose transporter type 4 (GLUT4), which leads to an increase in insulin sensitivity (Figure 3).

Metformin is mainly indicated in the management of T2DM, though it could be effective in the management of polycystic ovary syndrome and gestational diabetes.^32^ Metformin improves peripheral insulin sensitivity mainly in T2DM patients with obesity.^32^ It has been shown that metformin is regarded as a first‐line drug in treating T2DM patients when glycated hemoglobin (HbA1c < 7 mg/dL), and be used as monotherapy or in combination with other antidiabetic agents.^32^

Furthermore, metformin has pleiotropic properties like anti‐inflammatory and antioxidant effects, thereby reducing the risk of diabetic complications.^38^, ^39^ Metformin has been shown to exert an antineoplastic action in several types of tumors. Furthermore, these experimental data were also supported by clinical studies.^40^ Metformin potentiates the antitumor activity of MEK‐Is in human *LKB1*‐wild‐type NSCLC cell lines, through GLI1 downregulation and by reducing the NF‐jB (p65)‐mediated transcription of MMP‐2 and MMP‐9.^40^ A multicenter, open‐label phase II study showed that a combination of metformin with erlotinib as second‐line therapy in patients with stage IV non‐small‐cell lung cancer was effective in patients with lung cancer.^41^ Moreover, metformin exerts a cardioprotective effect through the improvement of the myocardial energy metabolic status by modulation of glucose and lipid metabolism, the attenuation of oxidative stress and inflammation, and the inhibition of myocardial cell apoptosis, leading to reduced cardiac remodeling and preserved left ventricular function.^42^ When compared with other antihyperglycemic medications, metformin has been demonstrated to be safe and to lower morbidity and mortality for heart failure.^42^ Furthermore, in addition to the protective action on the pathophysiology of AS, a protective action of metformin was observed in acute nondiabetic patients with acute myocardial infarction undergoing coronary artery bypass grafting (CABG).^43^ Metformin therapy might ameliorate cardiovascular outcomes by reducing inflammatory parameters, sodium−glucose cotransporter 2 (SGLT2), and leptin levels, and finally improving SIRT6 levels in patients with acute myocardial infarction treated with CABG.^43^ Metformin plays a role as a pleiotropic effector of adipocytes' inflammatory and metabolic functions. Interestingly, all of these metabolic and anti‐inflammatory effects ameliorated cardiac performance and clinical outcomes in T2DM patients. Thus, metformin could regulate the metabolic effectors linked to coronary endothelial dysfunction and restenosis, after coronary revascularization in T2DM patients.^43^

The use of metformin is associated with some adverse effects, including gastrointestinal disorders like diarrhea, nausea, vomiting, abdominal pain, and loss of appetite. However, prolonged use of metformin is associated with weight loss, B12 and folate deficiency, and the risk of peripheral neuropathy and cognitive impairment. Metformin has multiple benefits for health beyond its antihyperglycemic properties. Lactate‐mediated, mild, metabolic acidosis may thus drive some metformin‐mediated appetite suppression. Metformin has been shown to increase secretion of the weight‐loss‐promoting incretin GLP‐1, and the anorectic hormone peptide YY. A putative additional mechanism by which metformin may suppress appetite through action in the GI tract includes alteration of bile acid absorption through interaction with farnesoid X receptor. The mechanism of B12 and folic acid deficiency induced by prolonged metformin therapy is related to the disturbances in intestinal absorption of B12 and folic acid. Metformin promotes intestinal mobility disorders leading to bacterial overgrowth and/or it may alter intrinsic factor secretion which is essential to carry B12 for absorption from ileum.^44^ In addition, metformin displaces calcium in the ileal surface membrane, leading to disruption in intestinal calcium‐dependent B12 intrinsic factor uptake. Folate‐dependent target proteins are inhibited by metformin leading to deficiency of folic acid.^44^

Metformin therapy has a low risk of hypoglycemia because it does not stimulate insulin secretion.^45^, ^46^ A rare but serious adverse effect related to metformin use is lactic acidosis, which developed due to a reduction in the use of lactate due to an inhibited gluconeogenesis process. Lactic acidosis does not develop in all metformin patients, but only in those with heart failure, renal impairment, and alcohol consumption. Metformin toxicity due to overdosing leads to hypoglycemic and lactic acidosis, which are treated by hemodialysis.^47^ Regarding drug−drug interaction, metformin had little interaction with other drugs because it was not metabolized and was excreted unchanged by the kidney. However, some drugs like cimetidine, topiramate, ronalazine, and cephalexin increase the risk of developing lactic acidosis by competing with metformin's renal excretions. As well, some drugs like aspirin and antidiabetic agents increase the risk of hypoglycemia when used with metformin.^48^, ^49^ Furthermore, metformin use is contraindicated in patients with renal impairment when the glomerular filtration rate is less than 30 mL/min/1.73. In addition, metformin is contraindicated in patients with severe heart failure, metabolic acidosis, and hepatic dysfunction. However, metformin use is safe during pregnancy and lactation.^50^

## 3 METFORMIN AND AS

**Figure 4:**
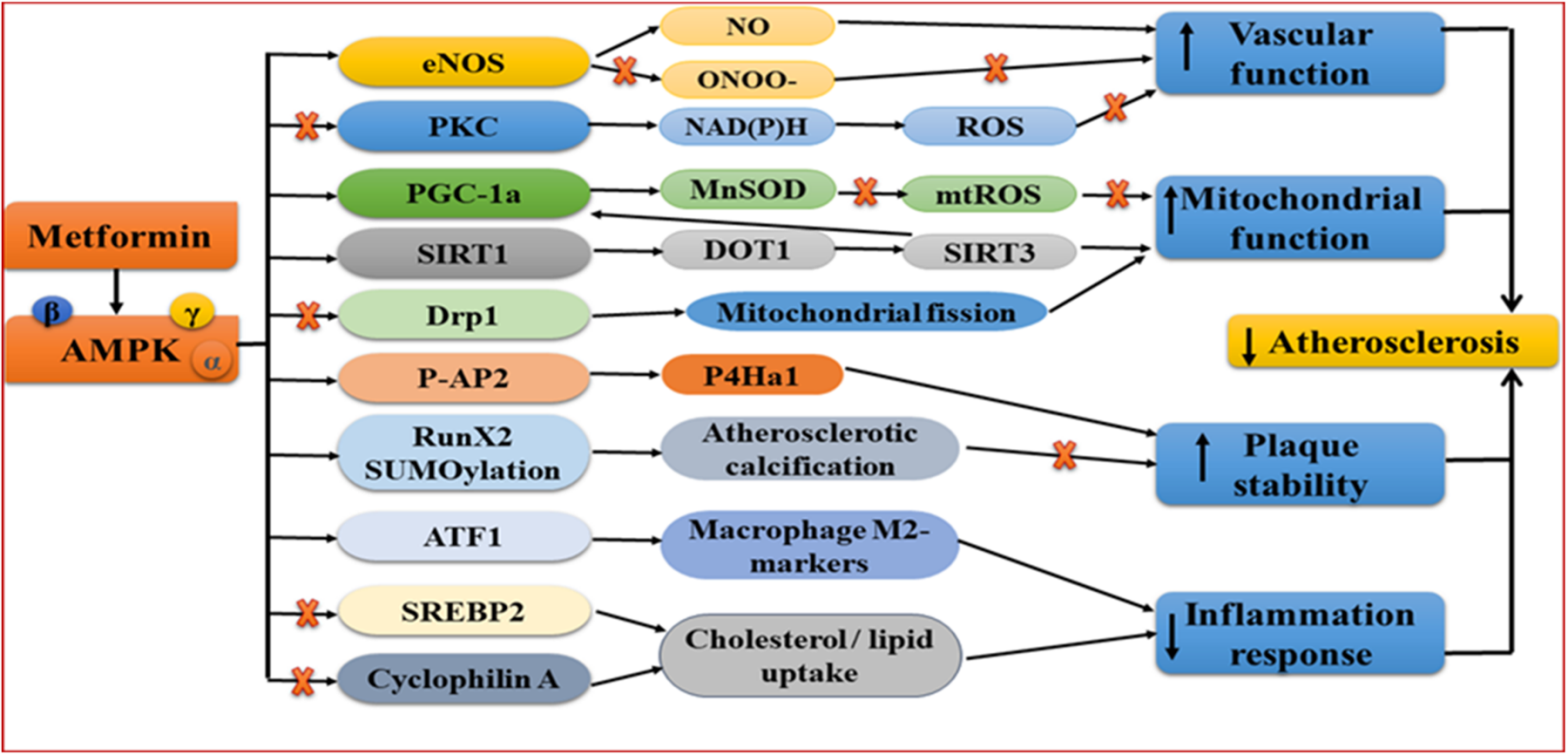
Metformin‐induced AMPK downstream different molecular cascades: Metformin‐induced AMPK improves vascular function through activation of endothelial nitric oxide synthase (eNOS) and protein kinase C (PKC). Metformin improves mitochondrial function via activation of silent information regulator 1 (SIRT1), peroxisome proliferator‐activated receptor gamma coactivator 1 alpha (PGC‐1α), and dynamin‐related protein 1 (Drp1). Metformin maintains plaque stability via activation of adipocyte fatty acid binding protein 2 (AP2) and runt‐related transcription factor 2 (RUNX2). Metformin reduces inflammation response by stimulating activating transcription factor 1 (ATF1) and inhibition of sterol regulatory element binding protein 2 (SREBP2). AMPK, adenosine monophosphate protein kinase.

Regarding the potential effect of metformin on AS, it has been reported that metformin, through activation of AMPK, induces the expression of heme‐oxygenase 1 (HO‐1) and anti‐inflammatory cytokines in endothelial cells. AMPK attenuates inflammatory changes, macrophage activation, and oxidative stress in AS.^51^ Metformin attenuates atherosclerotic plaque formation by inhibiting the differentiation of monocytes into macrophages through an AMPK‐dependent mechanism and slows the progression of AS by suppressing the inflammatory response and activation of macrophages in rabbit vascular endothelial cells.^52^, ^53^ Notably, metformin controls lipid homeostasis and inhibits cholesterol uptake in oxidative stress‐induced AS. Angiotensin II‐mediated dyslipidemia in rodents is regulated by metformin‐induced AMPK. Due to the inhibition of inflammation and preservation of vascular and mitochondrial functions, metformin‐induced AMPK downstream triggers various molecular cascades that result in plaque stability in AS.^54^ Therefore, metformin‐induced AMPK downstream different molecular cascades leading to plaque stability through inhibition of inflammation and maintaining vascular and mitochondrial functions in AS (Figure 4).

Metformin has been shown in clinical studies to protect against the development and progression of AS. For example, a double‐blind, placebo‐controlled clinical trial illustrated that metformin treatment in diabetic patients reduced AS risk significantly compared to placebo.^55^ In a randomized controlled clinical trial involving 50 diabetic patients with HIV and metabolic syndrome, metformin treatment reduced atherosclerotic plaque progression and reduced mortality in diabetic patients with AS.^56^ Compared to other antidiabetic agents with similar glycemic control, metformin has a vasculoprotective effect on cardiovascular morbidity and mortality. It also has pleiotropic properties, including anti‐inflammatory and antioxidant effects, which can lower the risk of AS. Originally, metformin slows the development of AS and the angiotensin II‐induced vascular smooth muscle proliferation. Metformin has a vasculoprotective effect against AS in T1DM and T2DM, according to recent clinical studies.^57^

**Figure 5:**
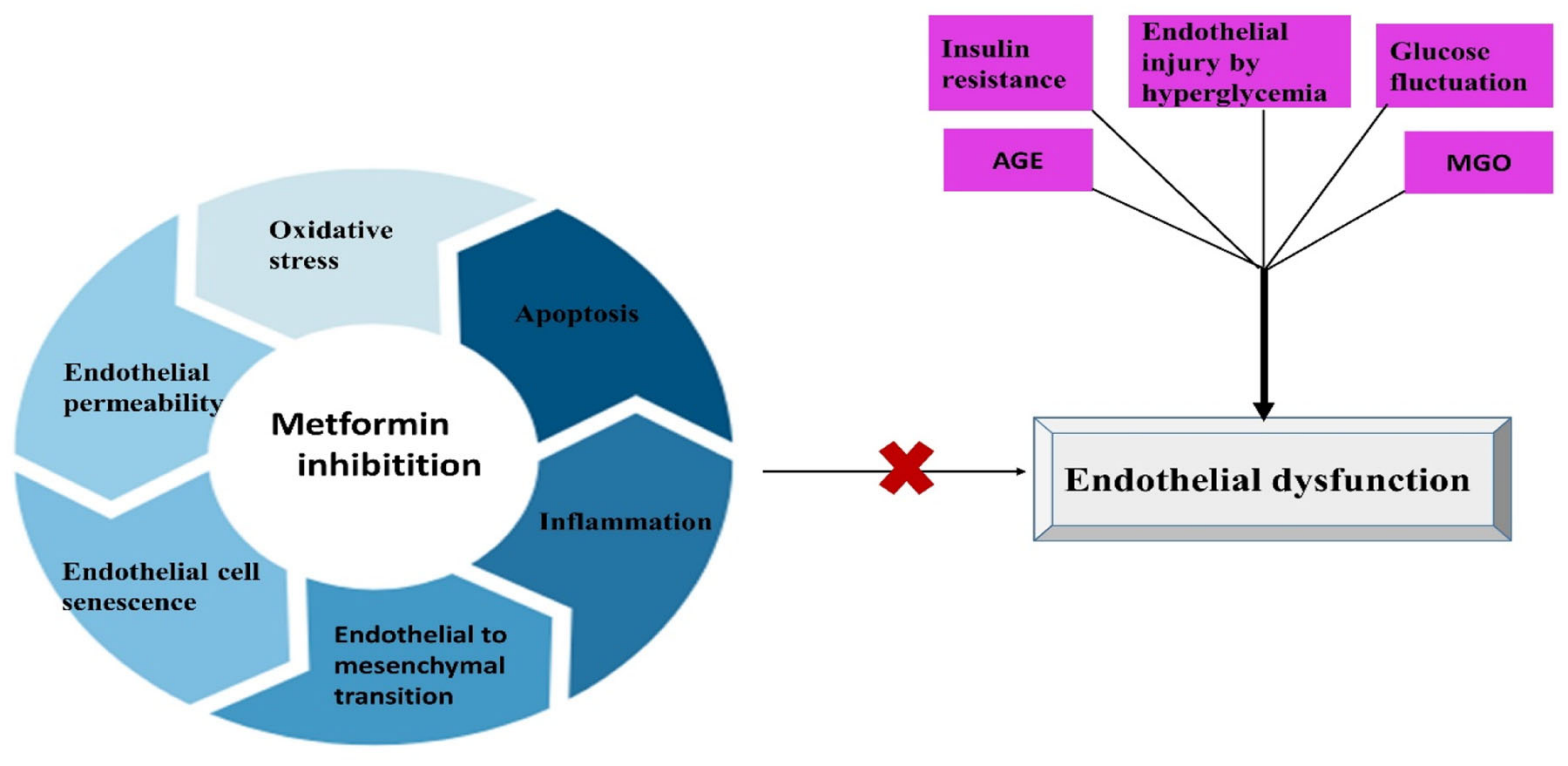
Role of metformin against the development of endothelial dysfunction: Endothelial injury by hyperglycemia, insulin resistance, glucose fluctuation, advanced glycation end‐products (AGEs), and methylglyoxal (MGO) lead to endothelial dysfunction. Metformin inhibits oxidative stress, endothelial permeability, endothelial cell senescence, apoptosis, inflammation, and endothelial−mesenchymal transition.

Endothelial dysfunction is thought to be a precursor stage in the development of AS, resulting in impaired vasodilation, increased oxidative stress and inflammation, endothelial hyperpermeability, and leukocyte adhesion with the progression of endothelial senescence.^54^ Impaired expression of endothelial glycocalyx is associated with endothelial dysfunction and is thought to be the first stage in the development of AS and can abrogate the development of AS through modulation of endothelial dysfunction and expression of glycocalyx.^58^, ^59^ Therefore, metformin, through attenuation of the development of endothelial dysfunction, may prevent the progression of AS (Figure 5).

**Figure 6:**
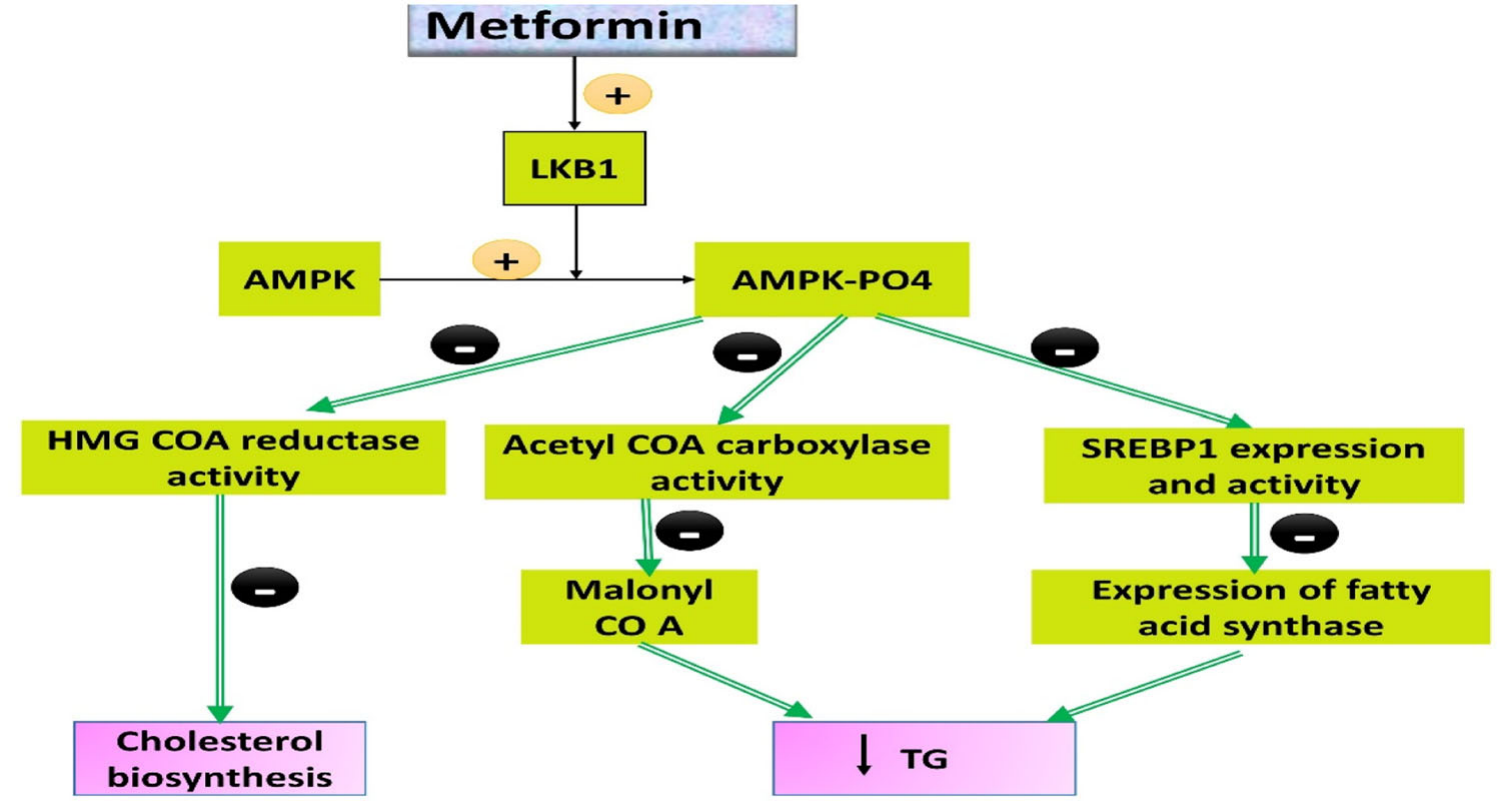
Metformin inhibits biosynthesis of cholesterol and triglyceride: Liver kinase B1 (LKB1) promotes activation and phosphorylation of adenosine monophosphate protein kinase (AMPK). Metformin inhibits the expression of hydroxymethylglutaryl CoA (HMG CoA) reductase with subsequent inhibition of cholesterol biosynthesis. Metformin reduces the expression of sterol regulatory element binding protein 1 (SREBP1), fatty acid synthase, and acetyl CoA carboxylase while reducing the synthesis of triglyceride (TG) and very low‐density lipoprotein (VLDL).

As reported, dyslipidemia is considered a cornerstone in the development of AS, and metformin may play a role in the treatment of dyslipidemia and its complications.^60^ Metformin treatment significantly reduced body weight, cholesterol, and non‐HDL cholesterol in kids with metabolic syndrome, according to a retrospective study involving 217 participants.^61^ Another research found that metformin monotherapy can lower IR, prevent LDL glycation, and promote weight loss. These actions all help to improve lipid profiles.^61^ As a result, metformin prevents the production of triglycerides and cholesterol through activating AMPK. The liver kinase B1 enzyme is important for AMPK activation, cholesterol regulation, and atherosclerotic plaque stability (Figure 6 adopted from https://www.ebmconsult.com/articles/metformin-glucophage-diabetes-lipid-cholesterol-lowering, 2015).

Furthermore, metformin increases the release of GLP‐1, which is an insulinotropic hormone that induces weight loss and decreases food intake. Metformin‐induced increment in GLP‐1 is mediated by inhibition of dipeptidyl peptidase 4 (DPP4), which is an enzyme involved in the degradation of GLP‐1.^62^ Additionally, metformin modulates gut microbiota, leading to increased expression and release of GLP‐1 and it has been shown that GLP‐1 reduces AS pathogenesis through modulation of foam cell formation. Notably, GLP‐1 receptors are highly expressed in monocytes, vascular smooth muscle cells, and endothelial cells, and relatively low expressed in human foam cells and macrophage.^63^, ^64^ An experimental study illustrated that GLP‐1 suppresses ox‐LDL‐mediated foam cell formation through downregulation of the expression of acyl‐CoA cholesterol acyltransferase 1 (ACAT1) in macrophages.^64^ A previous study found that GLP‐1 receptor agonists and other incretin‐based therapies for T2DM patients were associated with a low risk of developing AS, and their vasculoprotective effects were related to vasodilation, the prevention, and development of endothelial dysfunction, lipid homeostasis, and antiproliferative and anti‐inflammatory effects.^65^ Marquis‐Gravel and Tardif revealed that the use of GLP‐1 receptor agonists in T2DM patients was associated with good cardiovascular outcomes regarding AS risk, and these findings suggest that metformin may have an indirect protective effect against the development of AS by increasing GLP‐1 levels.^66^

## 4 EFFECT OF METFORMIN ON GROWTH DIFFERENTIATION FACTOR 15 (GDF15)

GDF15, a member of the TGF family, acts as a stress‐responsive cytokine that is activated during the atherosclerotic process and other cardiovascular complications, has antioxidant and anti‐inflammatory effects towards inflammatory disorders, and its level is positively correlated with cardiovascular risk factors and AS.^67^, ^68^ Al‐Kuraishy et al. observed that metformin increases the expression and release of GDF15, which has a protective effect against thrombosis and inflammation in T2DM patients, and they also reported that increasing the circulating GDF15 level in AS and T2DM patients could be a compensatory mechanism to mitigate and overcome oxidative stress and inflammatory disorders.^69^ As well as Ackermann et al. observed, GDF15 regulates lipid homeostasis, autophagy, and ox‐LDL expression in human macrophages.^70^ In contrast, through regulating the synthesis of interleukin 6, GDF15 deficiency lowers the inflammatory response to arterial injury that causes AS.^71^ However, GDF15 has a protective role against the development and progression of AS by inhibiting the expression of adhesion molecules and macrophage accumulation in mice.^72^ Therefore, GDF15 is regarded as an acute phase modulator of the inflammatory response during vascular injury.^73^ GDF15 deficiency in mice increases the vulnerability of atherosclerotic plaques to rupture, and these findings proposed that metformin could reduce atherosclerotic plaque complications by increasing the release of anti‐inflammatory GDF15.^72^

## 5 EFFECT OF METFORMIN ON TRIMETHYLAMINE N‐OXIDE (TAMO) AND GUT MICROBIOTA

Interestingly, the human microbiome, mainly gut microbiota, can translocate into the systemic circulation and colonize atherosclerotic plaques, leading to critical complications. In experimental mice, gut microbiota metabolites, mainly TAMO, induce the development of AS through impairment of cholesterol metabolism and induction of thrombosis.^74^ LiDy et al. observed that gut microbiota is a novel participator in the development and progression of AS.^75^

**Figure 7:**
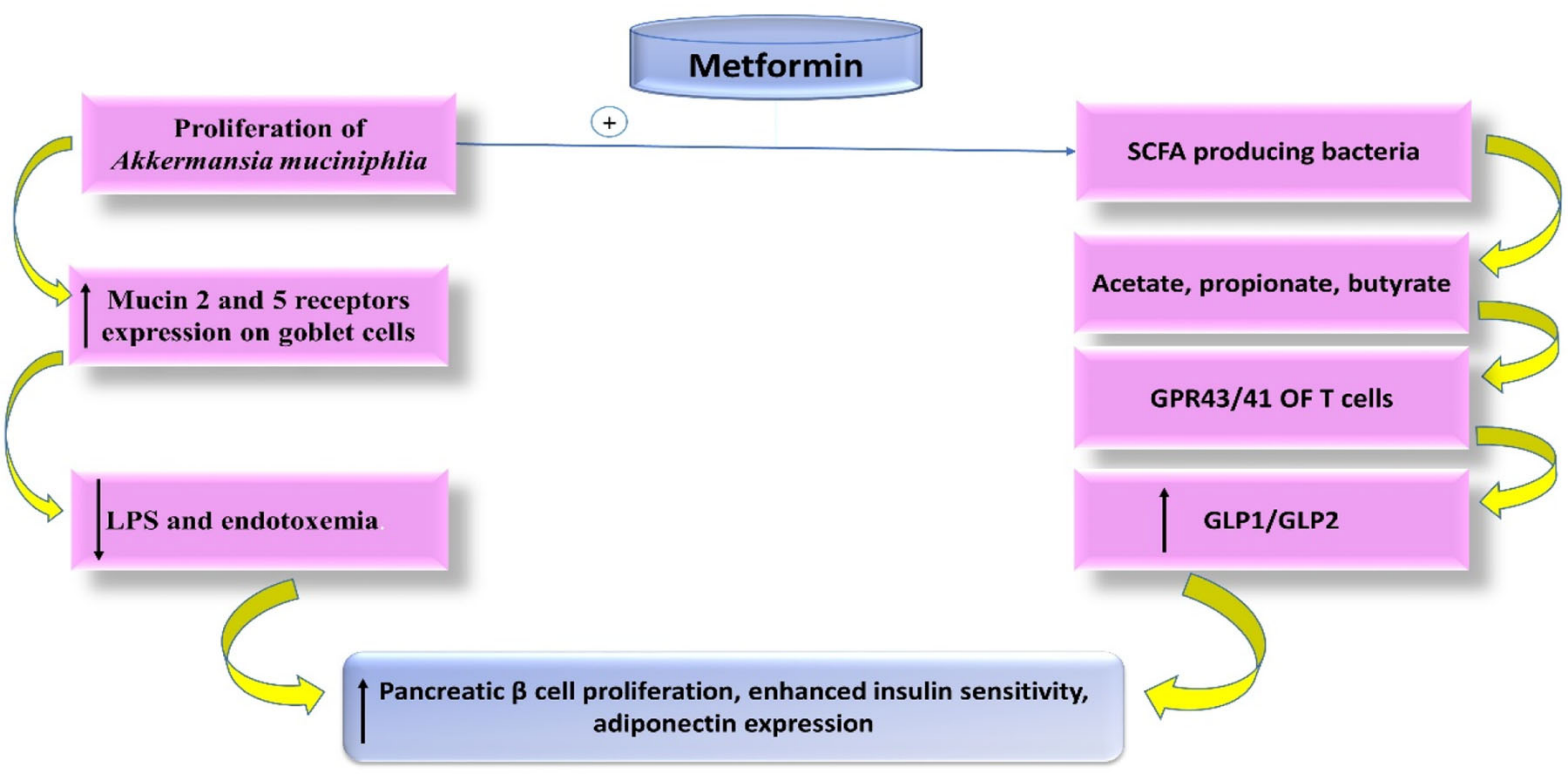
Effect of metformin on gut microbiota: Metformin promotes the proliferation of *Akkermansia muciniphlia*, which increases expression of mucin 2 and 5 receptors on goblet cells, leading to reduction of circulating LPS and effect of endotoxemia. As well, metformin increases the expression of short‐chain fatty acid (SCFA), producing bacteria that produce butyrate, propionate, and acetate. These bacterial metabolites activate G‐protein coupled receptor 43/41 (GPR43/41) on L‐cells leading to the release of GLP‐1 and GLP‐2, which enhance insulin sensitivity. GLP‐1, glucagon‐like peptide‐1.

Gut dysbiosis with an abundance of *Eubacterium* and *Roseburia* has been linked to an increased risk of AS development via the production of toxic metabolites and the induction of vascular inflammation, and the circulating LPS of the Gram‐negative gut microbiota activates TLR4 and CD14, leading to the release of proinflammatory cytokines and the development of endothelial dysfunction and AS.^76^ TAMO metabolite levels are increased in gut dysbiosis, causing vascular inflammation and AS through induction of inflammatory signaling pathways and the release of proinflammatory cytokines.^77^ Therefore, modulation of gut dysbiosis and inhibition of the release of bacterial toxic metabolites like TAMO could be an effective strategy against the development of AS. It has been shown that metformin inhibits TAMO production in mice.^78^ Metformin 250 mg/kg/day reduced TAMO levels in choline‐fed mice, lowering cardiovascular risk and normalizing gut dysbiosis and TAMO production.^78^ Recently, metformin reduced pro‐atherogenic TAMO in an experimental T2DM model via modulation of gut microbiota growth, and this might be a novel mechanism of the vasculoprotective effect of metformin against the development and progression of AS in T2DM via enhancement of insulin sensitivity and increased adiponectin expression.^78^ In sum, metformin promotes the proliferation of *Akkermansia muciniphlia*, which increases the expression of mucin 2 and 5 receptors on goblet cells, leading to a reduction of circulating LPS and the effect of endotoxemia. As well, metformin increases the expression of short‐chain fatty acid‐producing bacteria, which produce butyrate, propionate, and acetate. These bacterial metabolites activate G‐protein‐coupled receptors 43 and 41 (GPR43 and 41) on L‐cells, leading to the release of GLP‐1 and GLP‐2, which enhance insulin sensitivity^78^ (Figure 7 adopted from https://www.wjgnet.com/1948-9358/full/v10/i3/154.htm).

Taken together, metformin could be a promising therapy against the development and progression of AS through modulation of lipid homeostasis, inflammation, oxidative stress, GLP‐1, GDF15, and gut microbiota.

## 6 EFFECT OF METFORMIN ON Nr‐f2 IN AS

Nr‐f2 is a member of the basic leucine‐zipper family of transcription factors that conserve the cap'n'collar domain, counteracting oxidative stress via activation of the antioxidant response element.^50^ Nr‐f2 plays a critical role in the pathogenesis of AS, as it acts as anti‐atherogenic and pro‐atherogenic signaling. However, the main effect of Nrf2 is modulation of lipid homeostasis, macrophage polarization, inflammation, redox signaling, and foam cell formation.^50^

The pro‐atherogenic effect of Nr‐f2 is mediated by increasing non‐HDL cholesterol, liver adipogenesis, foam cell formation via upregulation of CD36, and expression of IL‐1.^79^ Nr‐f2 improves the expression of electrophile detoxification and antioxidant genes. Of interest, an experimental study confirmed that Nr‐f2 was pro‐atherogenic in mice only, despite its antioxidant effect.^80^ However, patients with insufficient Nr‐f2 activity are more likely to develop cardiovascular complications, as the antioxidant effect of Nr‐f2 alone is insufficient to protect against oxidative stress‐induced cardiovascular disorders, including AS and hypertension.^80^

**Figure 8:**
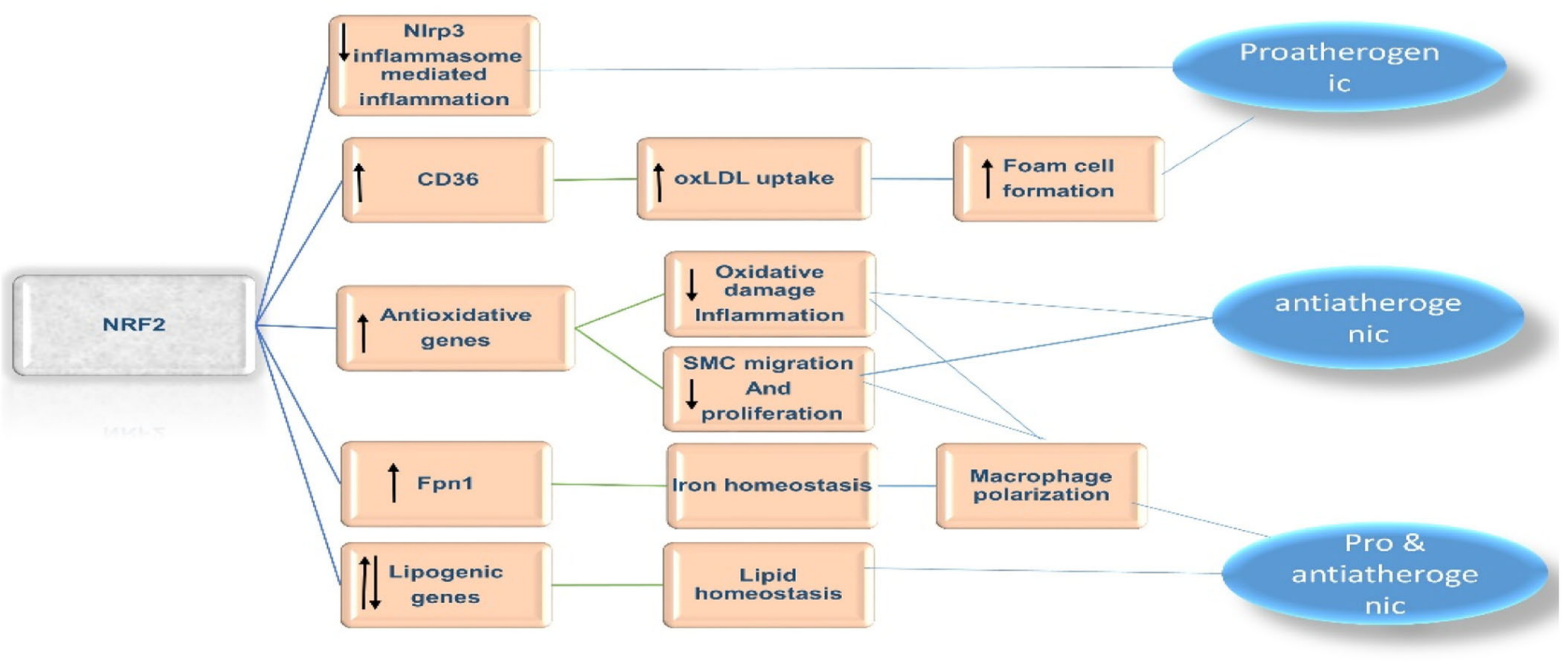
Role of nuclear erythroid‐related factor 2 (Nrf2) in atherogenesis: Nrf2 acts as an anti‐atherogenic by inhibiting oxidative stress. Nrf2 acts as a pro‐atherogenic by increasing lipid genes, activation of nod‐like receptor pyrin 3 receptor (NLRP3) inflammasome and foam cell formation. Nrf2 acts as pro‐atherogenic and anti‐atherogenic via dysregulation of iron homeostasis and macrophage polarization.

Though overexpression of Nr‐f2 may lead to detrimental effects and the acceleration of cardiovascular diseases, Remarkably, the short‐term effect of Nr‐f2 is beneficial, while the long‐term effect of Nr‐f2 might be detrimental.^81^ Nr‐f2 protects endothelial cells from hyperglycemia‐induced endothelial dysfunction and regulates macrophage activation and migration; thus, in Nr‐f2‐knockout mice, the macrophages were more activated with higher expression of proinflammatory genes. Furthermore, Nr‐f2 protects endothelial cells from hyperglycemia‐induced endothelial dysfunction and regulates macrophage activation and migration; thus, in Nr‐f2‐knockout mice, the macrophages were more activated with higher expression of proinflammatory genes. However, a lack of Nr‐f2 attenuates the development and progression of AS. This could be attributed to the reduced expression of the scavenger receptor CD36, which is involved in the uptake of LDL by macrophages.^80^ In conclusion, Nr‐f2 activity and function in AS seem to be complex, depending on the nature of Nr‐f2 in different cell types and the model system used. Thus, there is a controversial point regarding the potential role of Nr‐f2 in AS. Nr‐f2 acts as an anti‐atherogenic by inhibiting oxidative stress. Nrf2 acts as a pro‐atherogenic factor by increasing lipid genes, activation of the nod‐like receptor pyrin 3 receptor (NLRP3) inflammasome, and foam cell formation. Nrf2 acts as both pro‐ and anti‐atherogenic via dysregulation of iron homeostasis and macrophage polarization^80^, ^81^ (Figure 8 adopted from Mimura & Itoh^50^).

**Figure 9:**
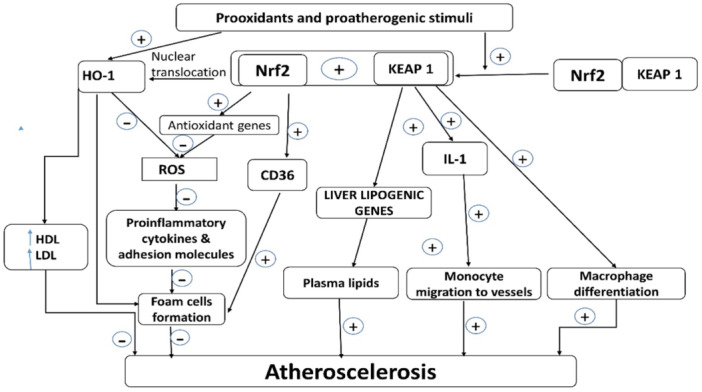
Differential effect of nuclear erythroid‐related factor 2 (Nrf2) in the development of atherosclerosis (AS): Nrf2 inhibits the generation of reactive oxygen species (ROS), the expression of proinflammatory cytokines, and the expression of adhesion molecules, with the subsequent inhibition of foam cell formation. As well, Nrf2 promotes the expression of antioxidant genes, increasing the expression of CD36 on the macrophages with successive inhibition of foam cell formation. These changes, mediated by HO‐1, prevent the development of AS. However, the direct effects of Kelch‐like ECH‐associated protein 1 (Keap1) lead to the polarization of macrophages to an inflammatory phenotype, promote the release of IL‐1 with an increment in monocyte migration, and increase the hepatic expression of genes related to lipid synthesis. These changes, mediated by Keap1, promote atherogenesis and the development of AS. HO‐1, heme‐oxygenase 1.

In fact, Nr‐f2 does not have a direct antioxidant effect; however, by activating HO‐1, it reduces ROS production, decreases the expression of macrophage scavenger receptors, and improves the LDL/HDL ratio.^82^ Kelch‐like ECH‐associated protein 1 (Keap1), a negative regulator of Nrf2 activity, increases macrophage activation, LDL, and monocyte migration into the vascular system, ultimately leading to AS.^82^ Nrf2 inhibits the generation of ROS, the expression of proinflammatory cytokines, and the expression of adhesion molecules, with the subsequent inhibition of foam cell formation. In addition, Nrf2 promotes the expression of antioxidant genes like glutathione (GPx), increasing the expression of CD36 on the macrophages with successive inhibition of foam cell formation. These changes, mediated by HO‐1, prevent the development of AS. However, the direct effect of Keap1 leads to the polarization of macrophages to an inflammatory phenotype, promotes the release of IL‐1 with an increment in monocyte migration, and increases the hepatic expression of genes related to lipid synthesis. These changes, mediated by Keap1, promote atherogenesis and the development of AS^82^ (Figure 9 adopted from Araujo et al.^82^).

HO‐1, a heme‐metabolizing enzyme that produces carbon monoxide (CO), ferrous iron, and biliverdin, which is then converted to bilirubin, is induced by stressful stimuli and acts as an adaptive defense mechanism to protect tissues in various diseases. HO‐1 has a protective effect against the development and progression of AS through the degradation of heme and the production of the antioxidant biliverdin.^83^ Keap1 inhibits Nrf2 activity when activated, exacerbating oxidative stress; thus, Keap1 inhibitors such as Ginsenoside reduce endothelial dysfunction and AS, and these findings illustrated that HO‐1 activators and Keap1 inhibitors may augment the atheroprotective effects of Nrf2.^84^

Nr‐f2 activators might be effective in various cardiovascular disorders; for example, sulforaphan prevents endothelial dysfunction and the progression of AS.^85^ As well, resveratrol increases the expression of Nrf2, which can attenuate oxidative stress and mitochondrial dysfunction. Therefore, resveratrol via the Nrf2‐dependent pathway may reduce the development and progression of AS.^86^ Interestingly, zinc promotes Nrf2 expression and could thereby be effective in treating cardiovascular disorders, including AS.^87^

Notably, metformin improves outcomes in mice with high‐fat diet‐induced obesity through the promotion of KEAP1 degradation and enhancement of Nrf2 expression and associated antioxidant pathways, including HO‐1.^88^ Rahimi et al. found that metformin reduced oxidative stress‐induced diabetic complications by modulating the Nrf2/Keap1 pathway in conjunction with herbal supplements or exercise.^89^ The primary mechanism by which metformin stabilizes Nr‐f2 and causes Keap1 to enter autophagy is thought to be AMPK.^90^ Metformin activates Nr‐f2 through AMPK‐dependent and AMPK‐independent mechanisms that decrease the risk of ischemic stroke.^35^ Pretreatment with metformin promotes the expression of Nrf2, HO‐1, and AMPK in the hippocampus of rats with experimental ischemic stroke.^35^ It has been shown that metformin mitigates atherosclerotic plaques via expression of Nrf2, HO‐1, activating transcription factor, and AMPK.^91^ A randomized clinical trial for 2 years of metformin use in nondiabetic patients showed that carotid intima‐media thickness was not significantly different from placebo. This might be due to early or subclinical AS and a lack of plaque assessment.^92^ Notably, Nrf2 activators when combined with metformin lead to greater protective effectiveness against the development of AS in diabetic patients.^93^ These observations suggested that metformin's activation of the Nrf2 pathway may reduce the risk of developing AS.

## 7 EFFECT OF METFORMIN ON KLF2 IN AS

**Figure 10:**
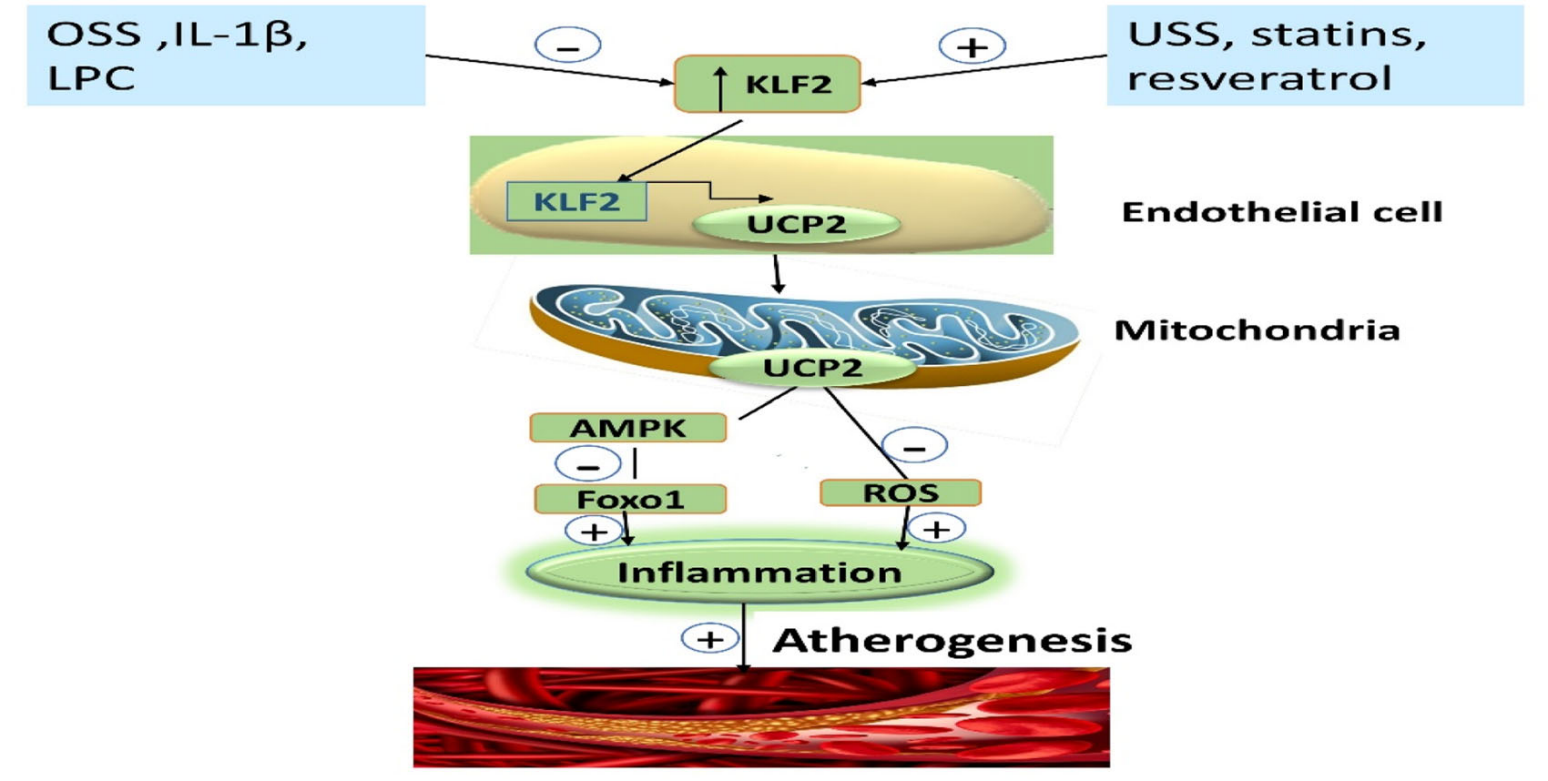
Role of Kruppel‐like factor 2 (KLF2) in atherogenesis: Unidirectional shear stress (USS), KLF2 activators statins and resveratrol promote expression of KLF2. Oscillatory shear stress (OSS), IL‐1β, and lysophosphatidylcholine (LPC) inhibit the expression of KLF2. KLF2 promotes the expression of uncoupling protein 2 (UCP2) in the endothelial cells, leading to the activation of mitochondrial function and release of AMPK, which inhibits forkhead box protein o1 (Foxo1). These changes mediated by KLF2 attenuate the generation of reactive oxygen species (ROS) and inflammation with subsequent inhibition of atherogenesis. AMPK, adenosine monophosphate protein kinase.

KLF2 is a central regulator of function and gene expression of monocyte/macrophage and endothelial cells, reduces vascular inflammation and progression of AS, regulates the proinflammatory functions of macrophage and endothelial cells, and activates expression of endothelial nitric oxide synthase (eNOS) and thrombomodulin. Thus, KLF2 has an atheroprotective effect, preventing the development of AS.^94^ Unidirectional shear stress, KLF2 activators statins, and resveratrol promote the expression of KLF2, but oscillatory shear stress, IL‐1, and lysophosphatidylcholine inhibit the expression of KLF2. KLF2 promotes expression of uncoupling protein 2 (UCP2) in the endothelial cells, leading to activation of mitochondrial function and the release of AMPK, which inhibits forkhead box protein 1 (Foxo1).^95^ These changes, mediated by KLF2, attenuate the generation of ROS and inflammation, with subsequent inhibition of atherogenesis.^95^ It has been reported that KLF2 levels were reduced in peripheral monocytes of patients with AS by about 30%, and enhancing KLF2 expression by specific activators mitigated endothelial dysfunction and AS through downregulation of proinflammatory genes.^96^ Therefore, KLF2‐based therapy could be a novel way in the management of AS (Figure 10 adopted from Luo et al.^95^).

Metformin‐induced KLF2 activation may lead to a beneficial effect in the amelioration of cardiovascular disorders, according to preclinical studies, as it has been shown that metformin attenuates endotoxemia‐induced endothelial dysfunction and proinflammatory response via activation of the AMPK/KLF2 axis. In vitro and in vivo findings revealed that metformin prevented LPS‐induced expression of adhesion molecules in endothelial cells and the release of proinflammatory cytokines via restoration of anti‐inflammatory KLF2.^96^, ^97^ Macrophage autophagy is an anti‐atherogenic process that maintains cellular lipid homeostasis by increasing cytosolic lipid catabolism, and improvement of this process by KLF2 may reduce the risk of AS.^97^ Metformin reduces HFD‐mediated AS and maintains plaque stability by increasing macrophage autophagy via KLF2 expression. Thus, metformin, through activation of the KLF2 pathway, is regarded as an anti‐atherogenic agent, preventing the development of AS.^97^ An in vitro study confirmed that KLF2 inhibits endothelial injury by activating autophagy and the expression of Nrf2. Therefore, this finding illustrated a potential crosstalk between KLF2 and Nrf2 during endothelial injury and the development of AS. As metformin, according to preclinical studies, is regarded as a dual activator of both KLF2 and Nrf2, it plays a critical role in the mitigation of inflammation during the atherogenic process.^98^

Furthermore, KLF2 expression in monocytes and endothelial cells was reduced in COVID‐19 patients due to the development of endothelial dysfunction, and KLF2 activators like atorvastatin ameliorated endothelial dysfunction in COVID‐19 patients.^99^ Grzegorowska et al. suggested a potential correlation between AS and COVID‐19 due to the development of endothelial dysfunction, inflammation, exaggeration of AngII, and plaque instability.^100^ The underlying molecular mechanism for this correlation could be related to dysregulation of the anti‐inflammatory and antioxidant KLF2/Nrf2 axis.^101^, ^102^ These findings suggest that metformin has a potential atheroprotective effect through activation of the KLF2 pathway.

Taken together, metformin is a functional therapeutic strategy against AS development and progression, mainly through modulation of the KLF2/Nrf2 axis.

## 8 CONCLUSION

AS is a progressive disease that interferes with blood flow, leading to tissue ischemia, mainly in the brain and heart, causing stroke and ischemic heart disease correspondingly. The underlying pathological conditions associated with AS progression are inflammation, oxidative stress, endothelial dysfunction, apoptosis, vascular proliferation, matrix degeneration, and neovascularization. Different signaling pathways, including Nrf2 and KLF2, are involved in the pathogenesis of AS. Nrf2 is a transcription factor that mediates various biological functions, including oxidative stress. KLF2 is a mechano‐sensitive transcription factor concerned with the regulation of endothelial functions and has anti‐inflammatory and antioxidant effects. Consequently, activation of these pathways by specific activators may reduce the progression and development of AS. Metformin, an insulin‐sensitizing drug used as a first‐line treatment for T2DM, increases the expression of Nrf2 and KLF2. Metformin has a vasculoprotective effect in the reduction of cardiovascular morbidities and mortalities compared to other antidiabetic agents, even with blood glucose control through Nrf2 pathway activation. Metformin has a potential atheroprotective effect through activation of the KLF2 pathway. Taken together, metformin is an effective therapeutic strategy against AS development and progression, mainly through modulation of the KLF2/Nrf2 axis. Preclinical and clinical studies are recommended in this regard.

## AUTHOR CONTRIBUTIONS

**Areej Turkistani**: Supervision; writing—original draft; writing—review and editing. **Haydar M. Al‐Kuraishy**: Conceptualization; data curation; formal analysis; funding acquisition; investigation; methodology; project administration; resources; software; supervision; validation; visualization; writing—original draft; writing—review and editing. **Ali I. Al‐Gareeb**: Conceptualization; data curation; formal analysis; funding acquisition; investigation; methodology; project administration; resources; software; supervision; validation; visualization; writing—original draft; writing—review and editing. **Athanasios Alexiou**: Conceptualization; data curation; formal analysis; funding acquisition; investigation; methodology; project administration; resources; software; supervision; validation; visualization; writing—original draft; writing—review and editing. **Marios Papadakis**: Conceptualization; data curation; formal analysis; funding acquisition; investigation; methodology; project administration; resources; software; supervision; validation; visualization; writing—original draft; writing—review and editing. **Mostafa M. Bahaa**: Conceptualization; data curation; formal analysis; funding acquisition; investigation; methodology; project administration; resources; software; supervision; validation; visualization; writing—original draft; writing—review and editing. **Salah Al‐Windy**: Conceptualization; data curation; formal analysis; funding acquisition; investigation; methodology; project administration; resources; software; supervision; validation; visualization; writing—original draft; writing—review and editing. **Gaber El‐Saber Batiha**: Conceptualization; data curation; formal analysis; funding acquisition; investigation; methodology; project administration; resources; software; supervision; validation; visualization; writing—original draft; writing—review and editing. **Areej Turjistani**: Revision; editing and proofreading. All authors contributed to the editing of the manuscript and performed extensive proofreading of the manuscript. All authors have read and approved the final manuscript.

## CONFLICT OF INTEREST STATEMENT

The authors declare no conflict of interest.

## Data Availability

All data generated or analyzed during this study are included in this published article.
